# High intraspecific variability and previous experience affect polyphenol metabolism in polyphagous *Lymantria mathura* caterpillars

**DOI:** 10.1002/ece3.10973

**Published:** 2024-02-09

**Authors:** Martin Volf, Alyssa M. Fontanilla, Suvi Vanhakylä, Tomokazu Abe, Martin Libra, Ryosuke Kogo, Roll Lilip, Naoto Kamata, Masashi Murakami, Vojtech Novotny, Juha‐Pekka Salminen, Simon T. Segar

**Affiliations:** ^1^ Biology Centre Czech Academy of Sciences Ceske Budejovice Czech Republic; ^2^ Faculty of Science University of South Bohemia Ceske Budejovice Czech Republic; ^3^ Department of Chemistry University of Turku Turku Finland; ^4^ Faculty of Science Chiba University Chiba Japan; ^5^ New Guinea Binatang Research Center Madang Papua New Guinea; ^6^ Graduate School of Agricultural and Life Sciences The University of Tokyo Tokyo Japan; ^7^ Agriculture and Environment Department Harper Adams University Newport UK

**Keywords:** chemical defenses, detoxification, flavonoids, frass, habituation, tannins

## Abstract

Polyphagous insect herbivores feed on multiple host‐plant species and face a highly variable chemical landscape. Comparative studies of polyphagous herbivore metabolism across a range of plants is an ideal approach for exploring how intra‐ and interspecific chemical variation shapes species interactions. We used polyphagous caterpillars of *Lymantria mathura* (Erebidae, Lepidoptera) to explore mechanisms that may contribute to its ability to feed on various hosts. We focused on intraspecific variation in polyphenol metabolism, the fates of individual polyphenols, and the role of previous feeding experience on polyphenol metabolism and leaf consumption. We collected the caterpillars from *Acer amoenum* (Sapindaceae), *Carpinus cordata* (Betulaceae), and *Quercus crispula* (Fagaceae). We first fed the larvae with the leaves of their original host and characterized the polyphenol profiles in leaves and frass. We then transferred a subset of larvae to a different host species and quantified how host shifting affected their leaf consumption and polyphenol metabolism. There was high intraspecific variation in frass composition, even among caterpillars fed with one host. While polyphenols had various fates when ingested by the caterpillars, most of them were passively excreted. When we transferred the caterpillars to a new host, their previous experience influenced how they metabolized polyphenols. The one‐host larvae metabolized a larger quantity of ingested polyphenols than two‐host caterpillars. Some of these metabolites could have been sequestered, others were probably activated in the gut. One‐host caterpillars retained more of the ingested leaf biomass than transferred caterpillars. The pronounced intraspecific variation in polyphenol metabolism, an ability to excrete ingested metabolites and potential dietary habituation are factors that may contribute to the ability of *L. mathura* to feed across multiple hosts. Further comparative studies can help identify if these mechanisms are related to differential host‐choice and response to host‐plant traits in specialist and generalist insect herbivores.

## Graphical abstract

We studied polyphenol metabolism in polyphagous caterpillars of *Lymantria mathura* (Erebidae, Lepidoptera) to explore mechanisms that may contribute to its ability to feed on various host‐plants. We detected pronounced intraspecific variation in polyphenol metabolism, an ability to excrete ingested metabolites and potential dietary habituation. Our results provide support for the hypothesis that generalists and specialists may have distinct metabolic strategies for dealing with secondary metabolites.

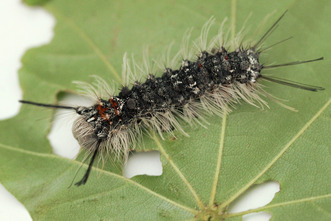

## 1 INTRODUCTION

Herbivorous insects show various levels of specialization in terms of their host‐choice (Ali & Agrawal, 2012). Most insect herbivores show some degree of specialization and feed on related plants within a genus or a family (Forister et al., 2015). The specialization of insect herbivores is often attributed to chemical variation among plants that have evolved a broad array of specialized metabolites that differ in their molecular structure, bioactivity, and distribution across the plant kingdom (Wetzel & Whitehead, 2020). Highly polyphagous generalist herbivores (an exception compared to most herbivorous insects) are ideal models for exploring how chemical variation across hosts affects herbivore preference, performance, and metabolism.

Theoretically, polyphagous insects should have “general” mechanisms to tolerate an array of plant chemical defenses (Ali & Agrawal, 2012). Herbivorous insects employ various strategies to cope with specialized metabolites of their hosts that can be broadly classified as: sequestration, detoxification, and passive excretion. Sequestration is typical for specialized herbivores that can use host‐plant metabolites as anti‐predator defenses or transform them into courtship pheromones (Fontanilla et al., 2022; Weller et al., 1999). In contrast, detoxification mechanisms can be found in both specialist and generalist herbivores. For example, the ability to detoxify glucosinolates is found across herbivore taxa with various levels of specialization and there are many variations, indicating convergence in outcome if not mechanism (Jeschke et al., 2016). Some detoxification mechanisms, such as glycosylation, are widespread among insects. Glycosylation helps detoxify metabolites like flavonoid aglycones that are found in many plants fed upon by both generalist and specialist insect herbivores (Salminen et al., 2004). Previous studies have suggested that generalist herbivores can, in some cases, possess a broad diversity of detoxification enzymes to metabolize a wide range of substrates (Heckel, 2014; Ramsey et al., 2010). However, such enzymes can be less efficient or fail to detoxify highly specialized chemical defenses (Johnson, 1999). Passive excretion of host‐plant metabolites without any modification has also been broadly documented among both specialists and generalists (Fontanilla et al., 2022; Salminen et al., 2004). Excretion may be an energetically favorable strategy in herbivores that do not utilize the metabolites of their hosts for their own benefit, because it does not require detoxifying host metabolites through glycosylation or comparable reactions (Salminen et al., 2004).

There can be large differences among conspecific individuals of generalist herbivores in their host‐choice (Jones & Agrawal, 2017). There is some evidence indicating that this variation in host‐choice can be linked to intraspecific variation in metabolism and midgut enzyme activity between conspecific generalist herbivores (Lazarević et al., 2017). Furthermore, host‐choice of herbivorous insects is not fixed and can be affected by dietary experiences (Zhou et al., 2021). Previous exposure to specialized metabolites can help insect herbivores overcome antifeedant or deterrent effects of plant tissue, and they can become habituated to the chemical defenses (Akhtar et al., 2003; Zhou et al., 2010). In caterpillars, diet composition also affects the metabolic response, with individuals feeding on different hosts showing a differential capacity to metabolize various specialized metabolites (Roy et al., 2016; Zhou et al., 2010). Previous experience can therefore affect how generalist caterpillars metabolize specialized metabolites following changing hosts. As even highly polyphagous generalists typically feed on chemically similar plants (Abe et al., 2021; Leong et al., 2022), one can expect the effect of previous experience to scale with chemical similarity among the hosts.

The rosy spongy moth, *Lymantria mathura* Moore (Erebidae, Lepidoptera), is a polyphagous insect herbivore of high ecological and economic importance. The species is native to the Russian Far East, India, northern China, and Japan (Zlotina et al., 1998). It feeds on a variety of broadleaf trees, including ca 20 different families (Volf et al., 2017; Zlotina et al., 1998). It is an important component of arboreal caterpillar communities in terms of biomass and number of individuals (Volf et al., 2017). It can reach outbreak levels and cause serious defoliation and damage to both forest and orchard trees (Kamata, 2002). The females have limited flight capacity and an important part of dispersal is realized by young caterpillars that use ballooning to reach new host‐plants (Zlotina et al., 1999). This dispersal strategy makes the final host of individual larvae largely unpredictable. Being able to process a variety of hosts with distinct defensive chemistry is key to larval survival.

Most trees utilized by *L. mathura* are rich in polyphenols. Polyphenols are widespread across plants and include metabolites such as flavonoids and tannins. Polyphenol defensive roles derive from at least three factors. First, polyphenols can show high oxidative activity that is activated by the high pH of the insect gut or by plant polyphenol oxidases released by cell lysis. Second, polyphenols can bind and precipitate nutritive proteins at low to neutral pH present in the oral cavity or in the gut of some insect species. Third, they also show activity resulting from degradation/hydrolysis products of polyphenols that may be accelerated by high pH or microbe action (Salminen, 2014; Salminen & Karonen, 2011). The relative importance of various types of polyphenol activity is intrinsically linked to their various fates upon ingestion by insect herbivores. For example, hydrolyzable tannins in an insect's gut can be hydrolyzed, oxidized, or adsorbed on the peritrophic membrane (Lahtinen et al., 2005). Importantly, the high pH found in the gut of lepidopteran larvae favors the oxidation of polyphenols and inhibits their protein precipitation functions (Salminen & Karonen, 2011). As a counter‐defense, insect herbivores can use ingested ascorbate to minimize the oxidative activity of polyphenols, detoxify flavonoid aglycones by glycosylation, or passively excrete the metabolites they ingested (Barbehenn et al., 2008; Salminen et al., 2004).

We performed an experiment with *L. mathura* larvae that were sampled and later fed with leaves from three tree species with different polyphenol compositions and activities. We analyzed leaf and frass polyphenols to quantify how larvae metabolized polyphenols contained in the leaves of their hosts. We then transferred a subset of these larvae across hosts (hereafter referred to as two‐host larvae) and measured how this affected their leaf consumption and polyphenol metabolism in comparison to one‐host larvae. Previous studies have shown relatively small intraspecific variability in frass composition among specialized caterpillars, some of which actively incorporate or modify the host metabolites (Fontanilla et al., 2022; Seifert et al., 2024). We expect that generalist *L. mathura* will process secondary metabolites differently. First, we expect that there will be relatively large intraspecific variation in polyphenol composition among frass samples, indicating pronounced intraspecific variation in how individual *L. mathura* caterpillars process these compounds. Second, we expect that many polyphenols will be excreted instead of being detoxified or sequestered. Third, we expect that previous dietary experience will affect polyphenol metabolism in two‐host larvae. And fourth, we expect that two‐host larvae will be able to digest less leaf biomass on their new host, with the mass consumed correlating negatively with chemical dissimilarity between the hosts. By performing these experiments, we aim to identify some of the mechanisms that may allow polyphagous caterpillars to overcome chemical dissimilarity among hosts.

## 2 METHODS

### 2.1 Experimental setup

We conducted the experiments in Tomakomai Experimental Forest (Hokkaido, Japan) in July 2015. We selected three locally dominant tree species commonly used by *L. mathura* as hosts: *Acer amoenum* Carr (Sapindaceae), *Carpinus cordata* Blume (Betulaceae), and *Quercus crispula* Blume (Fagaceae). We first sampled 20 leaves from 10 individuals of *A. amoenum*, 9 individuals of *C. cordata*, and 11 individuals of *Q. crispula* to explore variation in their polyphenol profiles. From this set of individuals, we then selected one individual of *A. amoenum*, two individuals of *C. cordata*, and one individual of *Q. crispula* and sampled 70–80 caterpillars of *L. mathura* from each species. Most of the caterpillars were in their 3rd instar by that time. We sampled caterpillars from two nearby individuals of *C. cordata* since this species is smaller in size, and one tree would not provide enough caterpillars. The sampling did not require any special permit. We continued feeding the caterpillars on the leaves of their original host tree individual until they reached the 4th instar. We transferred all *Carpinus* caterpillars to leaves from a single tree. We intentionally minimized the variation within hosts by using a single tree individual per species in the following feeding experiment. This allowed us to maximize our power to detect intraspecific differences in polyphenol metabolism among the caterpillars. We divided the subsequent experiment into two parts.

### 2.2 Phase I—Feeding on original hosts

We starved the larvae for 5 h before the experiment. We then enclosed the larvae individually into plastic containers and provided them with fresh leaves from their hosts, collected haphazardly from across the canopy to simulate the situation when different caterpillar individuals feed within different parts of the host tree. A subset of the same haphazardly collected fresh leaves from the same tree individuals was used for polyphenol analyses. We recorded the fresh weight of both sets of leaves. The caterpillars were allowed to feed for 24 h at 21°C under a standard summer 16:8 light regime. We collected caterpillar frass in 8‐h intervals to minimize the oxidation of polyphenols. Once we removed the larvae after 24 h, we dried the remaining leaves and weighed them. We also recorded the dry weight of leaves used for polyphenol analyses and used it to calculate fresh to dry weight ratio for leaves from each tree species. Using the fresh weight of leaves used for feeding the caterpillars and their dry weight after caterpillar feeding, we calculated the dry weight of the leaf tissue eaten by each caterpillar. We excluded all larvae that did not feed during Phase I (mainly because they started molting) from the subsequent experiments and analyses. This left us with 183 larvae successfully finishing Phase I and entering Phase II (Table 1).

**TABLE 1:** Number of *Lymantria mathura* caterpillars used in our analyses.

Phase I host	Number of caterpillars in Phase I	Phase II host	Number of caterpillars in Phase II
*Acer amoenum*	59	*Acer amoenum*	10
		*Carpinus cordata*	17
		*Quercus crispula*	17
*Carpinus cordata*	59	*Acer amoenum*	14
		*Carpinus cordata*	8
		*Quercus crispula*	9
*Quercus crispula*	65	*Acer amoenum*	17
		*Carpinus cordata*	14
		*Quercus crispula*	9

*Note*: We removed all larvae that did not feed or started molting during the respective experimental phase from the subsequent analyses, hence the difference in number of larvae between Phase I and Phase II.

### 2.3 Phase II—Changing hosts

We starved the caterpillars used in Phase I for 5 h before they entered Phase II. We then divided the larvae from each host into three groups of equal size. We kept feeding one group with the same host as before (one‐host caterpillars) while we transferred the remaining two groups to one of the other two tree species (two‐host caterpillars). We treated the larvae, collected leaves, and sampled frass the same as in Phase I. As in Phase I, we excluded all larvae that did not feed, started molting, or died during Phase II from the subsequent analyses. This resulted in 42 caterpillars fed with *Acer*, 39 caterpillars fed with *Carpinus*, and 35 caterpillars fed with *Quercus* in Phase II that were used in the final analyses (Table 1).

### 2.4 Chemical analyses

Polyphenols were extracted from ca 20 mg (with 0.01 mg accuracy) of freeze‐dried and homogenized material for 3 h using 1.4 mL acetone/water (80:20, v/v) as described in detail in Malisch et al. (2016). After extraction, centrifugation, and decanting, the extraction residue was re‐extracted for 3 h using 1.4 mL acetone/water (80:20, v/v). The two extracts were combined, evaporated to water phase and freeze‐dried. Freeze‐dried extracts were dissolved in 1 mL of Milli‐Q purified water and filtered with 0.2 μm polytetrafluoroethylene syringe filters. Leaf samples were 5 × diluted and frass samples were 2 × diluted with water before analysis. Samples were analyzed with ultra‐high performance liquid chromatography coupled with a photodiode array detector and triple quadrupole mass spectrometer (UHPLC‐DAD‐MS, Acquity UPLC® series, Waters Corporation, Milford, MA, USA). We used a 100 mm × 2.1 mm i.d., 1.7 μm, Acquity UPLC BEH Phenyl column (Waters Corp., Wexford, Ireland). Two solvents were used: acetonitrile (A) and 0.1% aqueous formic acid (B): 0–0.5 min, 0.1% A in B; 0.5–5.0 min, 0.1%–30% A in B (linear gradient); 5.0–6.0 min, 30%–35% A in B (linear gradient); 6.0–9.5 min, column wash and stabilization. The eluent flow rate was 0.5 mL/min. Negative ionization mode was used for MS analysis. Ultraviolet and MS data were recorded from 0 to 6 min. Capillary voltage was 2.4 kV, desolvation temperature 650°C, source temperature 150°C, desolvation and cone gas (N_2_) flow rates 1000 and 100 L/h.

We selected the most abundant and distinguishable compounds from the negative ion full scan MS chromatogram and ultraviolet chromatogram with detection wavelengths 349 nm for flavonoids and 280 nm for other compounds. We characterized the individual polyphenols based on their ultraviolet and MS spectra and retention times, and on the basis of our compound library including many of these specific compounds (Moilanen et al., 2013). For the quantification of the compounds, we used the TargetLynx tool (2017 Waters Inc. MassLynx software) to integrate the compound peaks from the extracted ion chromatograms obtained from the full‐scan MS data. Each compound was extracted from the full‐scan data by using the specific *m/z* value of the deprotonated molecules (e.g., *m/z* 933.3 for vescalagin) with a 0.8 Da mass window (e.g., *m/z* 932.9–933.7 for vescalagin), and integrated into its specific retention time window.

The selected compounds were quantified against external calibration curves obtained with our own standards (p‐coumaric acid for all coumaroyl quinic acids, 3‐O‐caffeoyl quinic acid for all caffeoyl quinic acids, vescavaloninic acid for vescavaloninic acid, vescalagin for vescalagin, castalagin for castalagin and cocciferin D2, geraniin for geraniin, 1‐O‐galloylglucose for 1‐O‐galloylglucose, kaempferol‐3‐O‐glucoside for all kaempferol glycosides, quercetin‐3‐O‐rhamnoside for all quercetin glycosides, apigenin‐7‐O‐glucoside for all apigenin glycosides, catechin for catechin and epicatechin, and procyanidin B2 for all PC dimers). The quantified polyphenols belonged to four polyphenol subgroups: (1) cinnamic acid derivatives, (2) flavonoids, (3) hydrolyzable tannins, and (4) proanthocyanidins. Second, we measured polyphenol oxidative activity and protein precipitation capacity as two of the main anti‐herbivore activities of polyphenols. We measured polyphenol oxidative activity by modified Folin–Ciocalteu assay following Salminen and Karonen (2011) using a Multiscan Ascent microplate reader (Labsystems and Thermo Electron Corporation). This method measures the phenolic content before and after alkaline oxidation in gallic acid equivalents. Protein precipitation capacity was measured with radial diffusion assay (Hagerman, 1987) by pipetting diluted samples to BSA‐agarose plate with a mixture of pentagalloylglucose and oenothein B as external standard. Both assays gave activities in mg/g dry weight.

### 2.5 Statistical analysis

To test our first hypothesis on the intraspecific variation in polyphenol composition among frass samples, we first explored the amount of variation in leaf polyphenols explained by the tree species identity with redundancy analysis (RDA) in the R package “vegan” (Oksanen et al., 2019). We used the data from 30 individuals of *A. amoenum*, *C. cordata*, and *Q. crispula*. We used tree species as explanatory variable, tested its significance with the Monte‐Carlo permutation test with 999 permutations, and computed the adjusted amount of variation explained. We applied a log + 1 transformation to the concentration of individual metabolites, the total contents of polyphenol subgroups, and polyphenol activities that were used as response variables. We performed separate analyses for (i) individual metabolites and (ii) total contents of polyphenol subgroups plus polyphenol activities. Second, we combined the leaf samples from the 30 tree individuals with frass data from Phase I and performed a principal components analysis (PCA) in the R package “stats” (R Core Team, 2022) to explore the variation in leaf and frass polyphenols. As before, we applied log + 1 transformation to the polyphenols used as response variables and performed separate analyses for (i) individual metabolites and (ii) total contents of polyphenol subgroups plus polyphenol activities.

To test our second hypothesis concerning the fate of ingested polyphenols, we explored the variation and fates of metabolized polyphenols in caterpillars fed with different tree species in Phase I. To do so, we used the difference between ingested and excreted polyphenols instead of simple concentrations as found in the frass. Based on the amount of the leaf tissue consumed, the amount of the frass produced, and the concentrations of polyphenols therein, we calculated the differences between ingested and excreted polyphenols as follows:

$$X=W_{\text{Leaf}}C_{\text{Leaf}}-W_{\text{Frass}}C_{\text{Frass}}$$

where *W* is dry weight in g and *C* is the concentration of polyphenols in mg/g in leaves and frass. Values close to zero indicate metabolites that were passively excreted (i.e., the ingested amounts were equal to the excreted amounts). Values above zero indicate metabolites partly lost in the gut, i.e., either those being taken up or those otherwise modified. Values below zero indicate metabolites at least partly produced in the gut by a modification of the ingested substrates. Using these data on metabolized polyphenols, we performed PCA and RDA as described above.

To test our third hypothesis on the effect of previous caterpillar experience on polyphenol metabolism, we compared the profiles of metabolized polyphenols among caterpillars with different dietary experiences. We divided the analyses according to the host species in Phase II, running separate analyses for caterpillars fed in this phase with *A. amoenum*, *C. cordata*, and *Q. crispula*. Similarly, as in the case of previous analysis with metabolized polyphenols, we used the differences between ingested and excreted polyphenols. We first performed PCA analyses followed by RDAs. In the RDAs, we used the previous host species as the explanatory variable, tested its significance with Monte‐Carlo permutation tests with 999 permutations, and computed the adjusted amount of variation explained. As in the case of all preceding analyses, we performed separate analyses for (i) individual metabolites and for (ii) total contents of polyphenol subgroups plus polyphenol activities that we used as response variables. In the case of analyses based on individual polyphenols, we included only metabolites that we previously recorded (>0.01 mg/g) in more than 25% of frass samples from the given host species in Phase I. We used this measure to focus solely on metabolites that are typical for the given host species, minimizing the effect of rare metabolites or possible contaminants.

To test our fourth hypothesis on the effect of previous dietary experience on the amount of digested biomass, we compared the amount of retained leaf biomass among the one‐ and two‐host larvae. We quantified retained leaf biomass as the difference between the dry weight of consumed leaf tissue and frass produced. We calculated the mean retained leaf biomass by one‐host larvae for each of the three tree species. We used this mean value as a baseline with which we compared the biomass retained by two‐host larvae. We compared the change in retained biomass between the larvae transferred to new hosts (two‐host larvae) using Mann–Whitney *U* tests. To place the decrease in retained biomass within the context of chemical similarity among the tree species, we calculated the Bray–Curtis dissimilarity index based on, (i) the presence and concentration of individual polyphenols and (ii) total contents of polyphenol subgroups plus polyphenol activities. All analyses we performed in R version 4.2.0 (R Core Team, 2022).

## 3 RESULTS

### 3.1 Leaf chemistry

**FIGURE 1:**
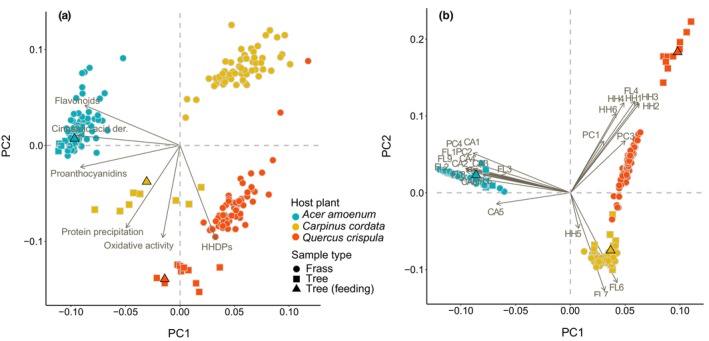
Variation in polyphenol subgroups and activities (a) and individual polyphenols (b) in leaves (squares) and frass (circles) of caterpillars fed with *Acer amoenum*, *Carpinus cordata*, and *Quercus crispula* in Phase I as visualized with PCA. The caterpillars were fed with a single tree individual per species (triangles). The first two unconstrained axes explained 90.2% of variability in polyphenol subgroups and activities and 76.6% of the adjusted variability in individual polyphenols. Arrows show polyphenol variables. The individual polyphenols include cinnamic acid derivatives (CA1–6), hydrolyzable tannins (HH1–6), flavonoids (FL1–9), and proanthocyanidins (PC1–4). Please see Table S1 for the full list of metabolite abbreviations.

We quantified 25 major polyphenols with a concentration ≥ 0.01 mg/g in the leaves and frass (Tables S1–S5) of the studied species. Many of the individual compounds were unique or abundant in one host species only, although all host species contained the same polyphenol subgroups. These included different cinnamic acid derivatives, flavonoid glycosides, hydrolyzable tannins, and proanthocyanidins and their building blocks, i.e., flavan‐3‐ols such as catechin and epicatechin (Table S1). Leaves from *A. amoenum* were high in flavonoid glycosides, such as apigenin glycosides, and in cinnamic acid derivatives, such as caffeoyl quinic acids. *Q. crispula* leaves were dominated by C‐glycosidic ellagitannins. These types of hydrolyzable tannins oxidize easily at high pH and cause high oxidative activity in the leaves of *Q. crispula*. Polyphenols from *C. cordata* leaves were dominated by the hydrolyzable tannin geraniin, followed by high levels of caffeoyl quinic acids and flavonol glycosides such as quercetin glycosides. Species identity explained 76.1% of the adjusted variability in polyphenol subgroups and activities (*F* = 47.17, *p* = .001) and 79.2% of adjusted variability in individual polyphenols (*F* = 56.34, *p* = .001) (Figure S1). When combined, the leaf and frass samples formed well‐defined clusters based on the host species identity (Figure 1). The variation among leaves from different tree individuals was generally similar or smaller than the variation among frass samples from different caterpillars, although the caterpillars were fed leaves from a single host individual (Table S2).

### 3.2 Intraspecific variation and fates of individual polyphenols (Phase I results)

**FIGURE 2:**
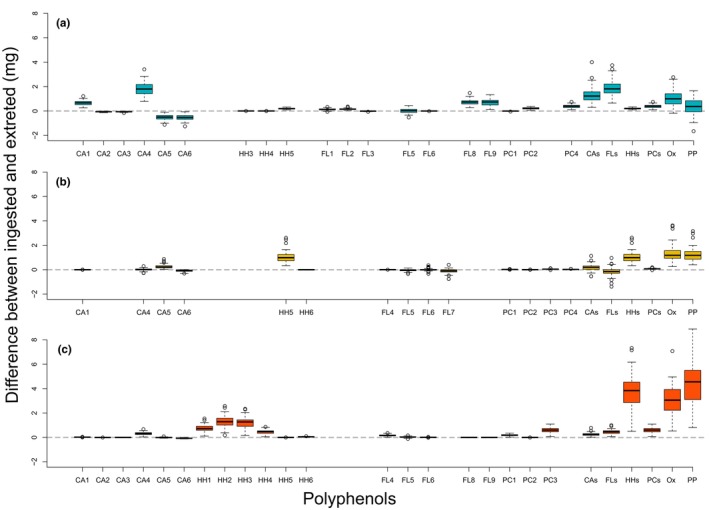
Differences between ingested and excreted individual polyphenols, their subgroups, and activities in caterpillars fed in Phase I with *Acer amoenum* (a), *Carpinus cordata* (b), and *Quercus crispula* (c). The differences are based on the amount of leaf tissue consumed, amount of frass produced, and concentrations of polyphenols therein. Values close to zero indicate metabolites that were passively excreted. Values above zero indicate metabolites partly lost in the gut, i.e., either those being taken up or those otherwise modified. Values below zero indicate metabolites at least partly produced in the gut by modification of ingested substrates. Only the metabolites that occurred in leaves or at least 25% of the frass samples from respective hosts are shown. The figure shows trends in individual cinnamic acid derivatives (CA1–6), individual hydrolyzable tannins (HH1–6), individual flavonoids (FL1–9), individual proanthocyanidins (PC1–4), total cinnamic acid derivatives (CAs), total hydrolyzable tannins (HHs), total flavonoids (FLs), oxitotal proanthocyanidins (PCs), oxidative activity (Ox), and protein precipitation capacity (PP). Please see Table S1 for the full list of metabolite abbreviations.

**FIGURE 3:**
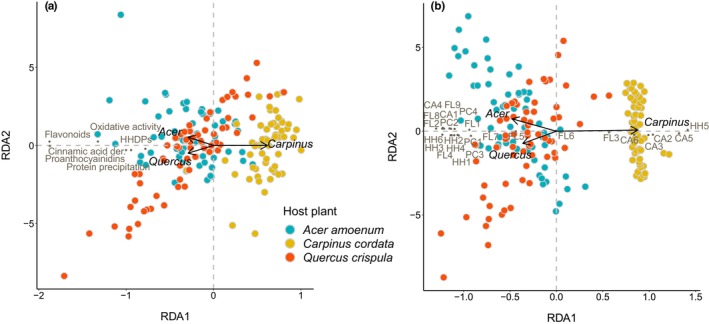
Variation in differences among ingested and excreted polyphenol subgroups and activities (a) and individual polyphenols (b) as metabolized by caterpillars fed with *Acer amoenum*, *Carpinus cordata*, and *Quercus crispula* in Phase I and analyzed with RDA. Host species identity explained 34.5% of the adjusted variability in polyphenol subgroups and activities (*F* = 48.91, *p* = .001) and 42.3% of the adjusted variability in individual polyphenols (*F* = 67.60, *p* = .001). Black arrows show explanatory variables, circles show frass samples, and points show polyphenol variables. The individual polyphenols include cinnamic acid derivatives (CA1–6), hydrolyzable tannins (HH1–6), flavonoids (FL1–9), and proanthocyanidins (PC1–4). Please see Table S1 for the full list of metabolite abbreviations.

Several polyphenols were passively excreted by the caterpillars, showing almost no difference between their ingested and excreted amounts (Figure 2). However, exceptions were found in all compound groups quantified and several individual polyphenols showed a positive difference between ingested and excreted amounts. Two cinnamic acid derivatives (caffeoyl quinic acids) showed a negative difference between their ingested and excreted amounts in caterpillars fed with *C. cordata*. A negative difference was also evident for the other types of cinnamic acid derivatives (coumaroyl quinic acids) found in *C. cordata*. Clusters of samples from caterpillars fed with *A. amoenum* and *Q. crispula* largely overlapped, while *C. cordata* samples formed a largely separated cluster when considering the differences between ingested and excreted polyphenols in Phase I (Figure S2). Most differences between ingested and excreted polyphenols showed a positive association with *A. amoenum* and *Q. crispula* and a negative association with *C. cordata*. Host species identity explained 34.5% of the adjusted variability in polyphenol subgroups and activities (*F* = 48.91, *p* = .001) and 42.3% of the adjusted variability in individual polyphenols (*F* = 67.60, *p* = .001) (Figure 3).

### 3.3 Trends after changing hosts (Phase II results)

**FIGURE 4:**
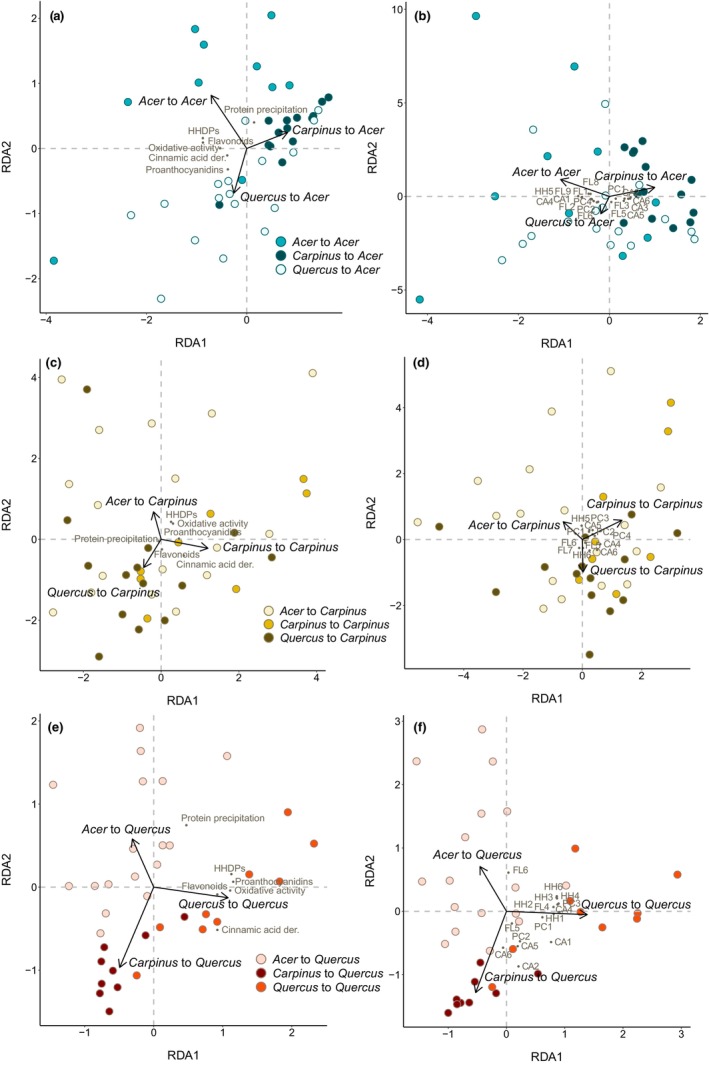
Variation in differences between ingested and excreted polyphenol subgroups and activities and individual polyphenols metabolized by caterpillars as explained by their previous experience and analyzed with RDA. In *Acer amoenum*, previous caterpillar experience explained 11.3% of the adjusted variability in polyphenol subgroups and activities (a; *F* = 3.54, *p* = .004) and 8.9% of the adjusted variability in individual polyphenols (b; *F* = 2.96, *p* = .006). In *Carpinus cordata*, previous experience did not affect polyphenol profiles in frass, explaining only 3.3% of the adjusted variability in polyphenol subgroups and activities (c; *F* = 1.66, *p* = .141) and 4.4% of the adjusted variability in individual polyphenols (d; *F* = 1.88, *p* = .073). Finally, in *Quercus crispula*, previous experience explained 42.9% of the adjusted variability in polyphenol subgroups and activities (e; *F* = 13.75, *p* = .001) and 36.7% of the adjusted variability in individual polyphenols (f; *F* = 10.97, *p* = .001). Black arrows show explanatory variables, circles show frass samples, and points show polyphenol variables. The individual polyphenols include cinnamic acid derivatives (CA1–6), hydrolyzable tannins (HH1–6), flavonoids (FL1–9), and proanthocyanidins (PC1–4). Please see Table S1 for the full list of metabolite abbreviations.

When we transferred the caterpillars to new hosts in Phase II, their previous experience affected how they metabolized polyphenols when subsequently reared on *A. amoenum* and *Q. crispula* (Figure 4, Figure S3). In *A. amoenum*, the previous host explained 11.3% of adjusted variability in polyphenol subgroups and activities (*F* = 3.54, *p* = .004) and 8.9% of adjusted variability in individual polyphenols (*F* = 2.96, *p* = .006) (Table S3). In *Q. crispula*, the samples from caterpillars that were fed with *Q. crispula* throughout (one‐host caterpillars), showed higher positive differences between ingested and excreted tissue for several individual polyphenols and oxidative activity when compared to two‐host caterpillars (i.e., the metabolites were lost in the gut). Previous experience explained 42.9% of adjusted variability in polyphenol subgroups and activities (*F* = 13.75, *p* = .001) and 36.7% of adjusted variability in individual polyphenols (*F* = 10.97, *p* = .001) (Table S4). Previous experience did not affect metabolized polyphenol profiles in caterpillars transferred to *C. cordata*, explaining only 3.3% of adjusted variability in polyphenol subgroups and activities (*F* = 1.66, *p* = .141) and 4.4% of adjusted variability in individual polyphenols (*F* = 1.88, *p* = .073) (Table S5).

**FIGURE 5:**
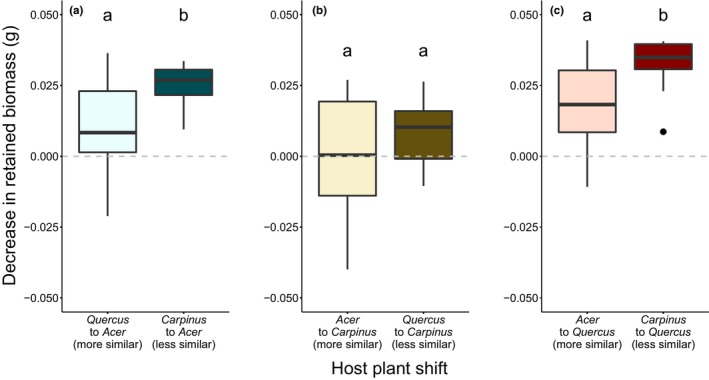
The relative decrease in retained biomass in caterpillars transferred to a new host. There was a larger decrease in retained biomass in caterpillars transferred to a host less chemically similar to the original one when the similarity between hosts was measured with Bray–Curtis similarity index based on the total contents of polyphenol subgroups and activities. The differences were significant in case of caterpillars transferred to *Acer amoenum* (*W* = 189, *p* = .005) and *Quercus crispula* (*W* = 120, *p* = .0183). The retained biomass refers to the difference between the dry weight of leaves ingested and dry weight of frass produced. The relative decrease in retained biomass was measured as the difference between biomass retained by individual caterpillars transferred to a new host and mean biomass retained by one‐host caterpillars.

The amount of retained biomass significantly decreased in caterpillars transferred from *C. cordata* to *A. amoenum* (*x* ^*2*^_(2)_ = 11.47, *p* = .003) and from *C. cordata* to *Q. crispula* (*x* ^*2*^_(2)_ = 12.72, *p* = .002) in comparison to one‐host caterpillars (Figure S4). The relative decrease in retained biomass positively correlated with chemical dissimilarity between the original and new host (Table S6) based on the total contents of polyphenol subgroups and activities in *A. amoenum* (*W* = 189, *p* = .005) and *Q. crispula* (*W* = 120, *p* = .0183) (Figure 5). We recovered a trend in the opposite direction when considering similarity based on presence and concentration of individual polyphenols (Figure S5).

## 4 DISCUSSION

In this study, we explored how highly polyphagous *Lymantria mathura* caterpillars deal with specialized metabolites of their hosts. Our results show large variability in polyphenol metabolism among individual caterpillars coupled with a significant effect of habituation. These results contribute to our understanding of how *L. mathura* maintains its varied diet and may have implications for other generalist insect herbivores.

### 4.1 Intraspecific variation

As we expected, there was large variability in frass composition and polyphenol metabolism among conspecific generalist caterpillars. Our results are in contrast to the recent results of Fontanilla et al. (2022) who studied highly specialized *Asota* tiger moths that feed on alkaloid‐rich *Ficus* hosts. Fontanilla et al. (2022) showed that frass composition converges across different *Asota* species despite them feeding on chemically distinct plants. Chemical convergence indicates that *Asota* caterpillars employ a relatively narrow array of strategies in terms of metabolizing the metabolites of their hosts (Fontanilla et al., 2022). Here, we found that frass polyphenol profiles formed well‐separated clusters depending on host identity, in some cases largely resembling those of leaves. At the same time, the variation in frass polyphenols was typically larger or similar to the variation in leaf polyphenols, even though the leaves came from multiple trees while only one tree per species was used for caterpillar feeding. Similarly large intraspecific variation in polyphenol metabolism has also been found in other lepidopteran larvae such as *Epirrita autumnata* (Geometridae), another important polyphagous defoliator of broadleaf trees (Salminen et al., 2004), even when fed with purified hydrolyzable tannins (Salminen, 2018; Salminen & Lempa, 2002). Large intraspecific variation has been also observed in *Lymantria dispar* (Barbehenn et al., 2009) and sawfly larvae (Lahtinen et al., 2005). Theoretically, such large metabolic variation among conspecific caterpillars can serve to generate a pool of individuals and phenotypes with different metabolic capacities that may be able to feed on different hosts (Jones & Agrawal, 2017; Lazarević et al., 2017). Such a bet‐hedging strategy may be especially important in polyphagous herbivores such as *L. mathura* that face large variation in chemistry across their potential hosts due to unpredictable encounters (Abe et al., 2021; Lazarević et al., 2017). However, further comparative studies are required to explore if there truly are any systematic differences in intraspecific variation in metabolisms between specialist and generalist herbivores. Even when feeding on a single host species, individuals of both generalist and specialist caterpillars face pronounced chemical variation both among conspecific hosts and within canopies of individual trees (Murakami et al., 2005; Volf et al., 2022).

### 4.2 Fates of individual polyphenols

When ingested by the caterpillars, individual polyphenols had mixed fates. Lepidopteran larvae are expected to have basic gut pH that causes oxidation of some of the most active polyphenols and isomerization of caffeoyl or coumaroyl quinic acids (Kim et al., 2018; Salminen et al., 2004). For this reason, the total caffeoyl quinic acid levels dropped from leaves to frass, while 5‐*O*‐caffeoyl quinic acid levels were much higher in frass than in the leaf diet, when fed with *A. amoenum* or *Q. crispula*. This was due to the isomerization of the three caffeoyl quinic acid isomers, thus causing increases in the content of the minor isomers and drops in the major isomers. These isomerization patterns allowed us to estimate the pH of the gut of *L. mathura* caterpillars. Using the foliar levels of the three caffeoyl quinic acids in the pH simulation of Kim et al. (2018), we can simulate the isomer ratios of these compounds across a range from pH 9.0 to 11.0 and compare those to ratios found in the actual frass. With all three host species, the average gut pH was estimated to be between pH 9.8 and 10.0, and most close to pH 9.9, a range of values similar to that found in other polyphagous caterpillars feeding on a variety of broadleaf trees (Kim et al., 2018).

High caterpillar gut pH affected the breakdown of ellagitannins, which are known to be one of the most oxidatively active group of polyphenols (Barbehenn et al., 2006; Moilanen & Salminen, 2008). Their contents, as with foliar oxidative activity, decreased significantly in the alkaline gut. This suggests the oxidative activation of these polyphenols in the gut instead of passive excretion, which is likely one of the mechanisms contributing toward the pronounced effects of ellagitannins and their oxidative activity on communities of caterpillars associated with broadleaf trees (Segar et al., 2017). Here, oxidation was especially pronounced when larvae were fed with *C. cordata* leaves, since its main ellagitannin, geraniin, lost >98% of its content from leaf to frass. This was surprising in comparison to loss of C‐glycosidic ellagitannin content in *Q. crispula* (such as vescalagin, castalagin, and vescavaloninic acid) that dropped by only 70%–87%, although they have 7–15 times higher oxidative activity in vitro than geraniin (Moilanen et al., 2016; Moilanen & Salminen, 2008). Similar calculations for the loss of oxidative activity from leaves to frass showed 29% loss for *Acer*, 53% loss for *Q. crispula*, and 72% loss for *C. cordata*. This suggests that the alkaline gut condition was not the sole driver of polyphenol oxidation, but that *C. cordata* leaves probably contained more oxidative enzymes, such as polyphenol oxidases, than did *Q. crispula* leaves (Kim et al., 2018). In turn, *C. cordata* leaves provided the best oxidative combination of defensive metabolites and enzymes, pointing to important synergies between content of specialized metabolites and enzymatic reactions.

Interestingly, protein precipitation capacity showed a strikingly different trend. It decreased by 97% in *C. cordata* and by 50% in *Q. crispula*, but even increased by 34% in *A. amoenum*. This could be due to the oxidative transformation of *A. amoenum* proanthocyanidins (condensed tannins) in the alkaline gut into even larger tannins with better protein affinity (Imran et al., 2021; Karonen et al., 2021). Alternatively, it could point to the accumulation of foliar proanthocyanidins in the frass while other leaf components were better digested and did not contribute to the frass mass. The accumulation of proanthocyanidins is supported by the oligomeric and polymeric procyanidin‐rich proanthocyanidins that we found in *Acer* frass, which were present in significantly higher levels than in the leaves (data not shown). The specific fate of these large tannins was presumably due to their active filtration through the gut into the frass. While on average 36% of the ingested *Acer* leaf mass was not excreted, this allowed the 34% increase in the content of protein precipitation capacity due to the readily excreted polymeric PC‐rich proanthocyanidins. This is in line with the low oxidative activity of such tannins at high pH (Barbehenn et al., 2006; Imran et al., 2021) and the apparently lower enzymatic activity in *A. amoenum* versus *Q. crispula* and *C. cordata* as discussed above.

The metabolic fate was different even within the compounds containing the same dihydroxysubstituted catechol ring (compounds CA4–CA6, FL4–FL7, and PC1–PC4) that is found in the above‐mentioned procyanidin‐rich proanthocyanidins. For instance, in the enzymatically active *C. cordata*, flavan‐3‐ol monomers and dimers (PC1–PC4) disappeared almost fully while the content of quercetin glycosides (FL5–FL7) with the same catechol unit dropped only slightly. These patterns also suggest compound‐specificity in the enzymatic oxidation in *Carpinus*‐fed larvae towards flavan‐3‐ol type rather than quercetin‐type moieties within the flavonoid‐type structures. In addition to alkaline and enzymatic oxidation, there are other fates for tannins and other polyphenols in insect herbivores. For instance, some hydrolyzable tannins are prone to hydrolysis in basic gut conditions (Lahtinen et al., 2005; Salminen, 2018; Salminen & Lempa, 2002). Hydrolysis, in general, can generate smaller metabolites that can penetrate the peritrophic membranes in the gut, and together with oxidation can cause oxidative stress to the herbivore via quinones and reactive oxygen species (Bernays, 1978; Salminen & Lempa, 2002). In this study, we did not look for specific hydrolysis products in the frass and did not analyze insects for possible hemolymph‐bound or other metabolites. However, the specific effects of small metabolites entering insect hemolymph are not yet fully elucidated. Salminen (2018) suggested that some of the effects could be even positive for the herbivore. Unfortunately, we lack data on the caterpillar performance to analyze if any of the metabolites could have affected the caterpillars either way. Analyzing these metabolites and their fates in the context of caterpillar performance and growth would therefore be the next important step towards understanding their effects in insect herbivores.

### 4.3 Trends after changing hosts

Previous dietary experience affected polyphenol metabolism, especially in the case of caterpillars transferred to *Q. crispula*. Previous dietary experience has been recorded to affect metabolism in other generalists, such as in *Spodoptera littoralis* where it led to transcriptomic readjustments that affected detoxification and nutrient takeup by the caterpillars (Roy et al., 2016). Theoretically, adjusting metabolism to the current host could be one of the mechanisms related to habituation, in addition to sensory changes that affect the herbivore's preference (Akhtar et al., 2003; Zhou et al., 2010). Here, caterpillars transferred from *A. amoenum* to *Q. crispula* showed a larger decrease in protein precipitation capacity and a smaller decrease in oxidative activity from leaves to frass than one‐host larvae fed continuously with *Q. crispula*. The caterpillars continuously fed with *Q. crispula* also generally metabolized larger amounts of ingested polyphenols than caterpillars transferred to this host. While some of these metabolites could be taken up (Salminen, 2018), others were probably activated in the gut, as also suggested by the high oxidative activity in these frass samples. Assuming that at least some of these reactions could be harmful to the caterpillars, our results suggest that the one‐host caterpillars did not necessarily deal with the polyphenols of their hosts more efficiently than two‐host caterpillars.

The one‐host caterpillars retained more biomass than the two‐host caterpillars. Interestingly, caterpillars that were transferred to the new diet from chemically similar hosts were able to retain more biomass than those transferred from the less similar ones when we considered the polyphenol subgroups and activities. Concentration of polyphenol subgroups and activities was shown to be important drivers of herbivore community composition across various systems, including those consisting of polyphagous generalist species (Abe et al., 2021; Segar et al., 2017). Our results are somewhat in line with these previous studies. However, data on caterpillar growth and survival would be needed to test if the observed differences in the retained biomass link to caterpillar performance. More details on transcription and the exact fates of individual metabolites would also help to test if any potential effects on caterpillar performance occur due to readjustments in detoxification mechanisms, or other forms of tolerance to current host metabolites or metabolites derived from hosts with similar chemistry (Roy et al., 2016).

## 5 CONCLUSIONS

In conclusion, we show that there are several mechanisms that can potentially contribute to the ability of *L. mathura* caterpillars to feed on various broadleaf hosts. These mechanisms involve pronounced intraspecific variation in polyphenol metabolism, the ability to excrete a large proportion of the ingested metabolites, and possible habituation of the larvae to their diet. A limitation of our study is that we lack data on growth and survival of caterpillars that would be fed with different hosts over a longer period of their life cycle. Therefore, we cannot relate directly the detected patterns to caterpillar performance. However, some of the patterns are similar to those found in other generalist herbivores while they contrast to the trends recovered in specialists (Fontanilla et al., 2022; Salminen et al., 2004; Seifert et al., 2024). As such, the differential metabolism of host‐plant metabolites may be one of the key factors promoting different responses to host‐plant traits among specialist and generalist insect herbivores (Ali & Agrawal, 2012). Further comparative studies will help identify the relative importance of individual mechanisms that promote the differential responses among herbivores. Finding these underlying mechanisms will help to understand some of the factors fuelling the plant‐insect arms race through reciprocal counter‐adaptations in plant defense and insect metabolism.

## AUTHOR CONTRIBUTIONS

**Martin Volf:** Conceptualization (equal); data curation (equal); formal analysis (equal); investigation (equal); methodology (equal); supervision (equal); visualization (equal); writing – original draft (lead); writing – review and editing (equal). **Alyssa M. Fontanilla:** Formal analysis (equal); visualization (equal); writing – original draft (equal); writing – review and editing (equal). **Suvi Vanhakylä:** Formal analysis (equal); investigation (equal); methodology (equal); writing – review and editing (equal). **Tomokazu Abe:** Investigation (equal); writing – review and editing (equal). **Martin Libra:** Investigation (equal); writing – review and editing (equal). **Ryosuke Kogo:** Investigation (equal); writing – review and editing (equal). **Roll Lilip:** Investigation (equal); writing – review and editing (equal). **Naoto Kamata:** Funding acquisition (equal); supervision (equal); writing – review and editing (equal). **Masashi Murakami:** Funding acquisition (equal); methodology (equal); project administration (equal); supervision (equal); writing – review and editing (equal). **Vojtech Novotny:** Conceptualization (equal); funding acquisition (equal); methodology (equal); supervision (equal); writing – original draft (equal). **Juha‐Pekka Salminen:** Conceptualization (equal); formal analysis (equal); funding acquisition (equal); investigation (equal); methodology (equal); supervision (equal); writing – original draft (equal); writing – review and editing (equal). **Simon T. Segar:** Conceptualization (equal); formal analysis (equal); methodology (equal); supervision (equal); writing – original draft (equal); writing – review and editing (equal).

## CONFLICT OF INTEREST STATEMENT

We declare that we have no conflict of interest.

## Supporting information


**Figure S1.** **–S5.**

**Table S1.**–**S6.**

## Data Availability

The data are available from Zenodo (https://doi.org/10.5281/zenodo.8211881).
