# Supplementary material for: High intraspecific variability and previous experience affect polyphenol metabolism in polyphagous Lymantria mathura caterpillars
Source: Ecol Evol. 2024 Feb 9;14(2):e10973. doi: 10.1002/ece3.10973 (PMC10857923; doi:10.1002/ece3.10973)
Supplement: Supplementary file 1 — Figure S1. –S5. [file ECE3-14-e10973-s001.pdf]

Supplementary Figures

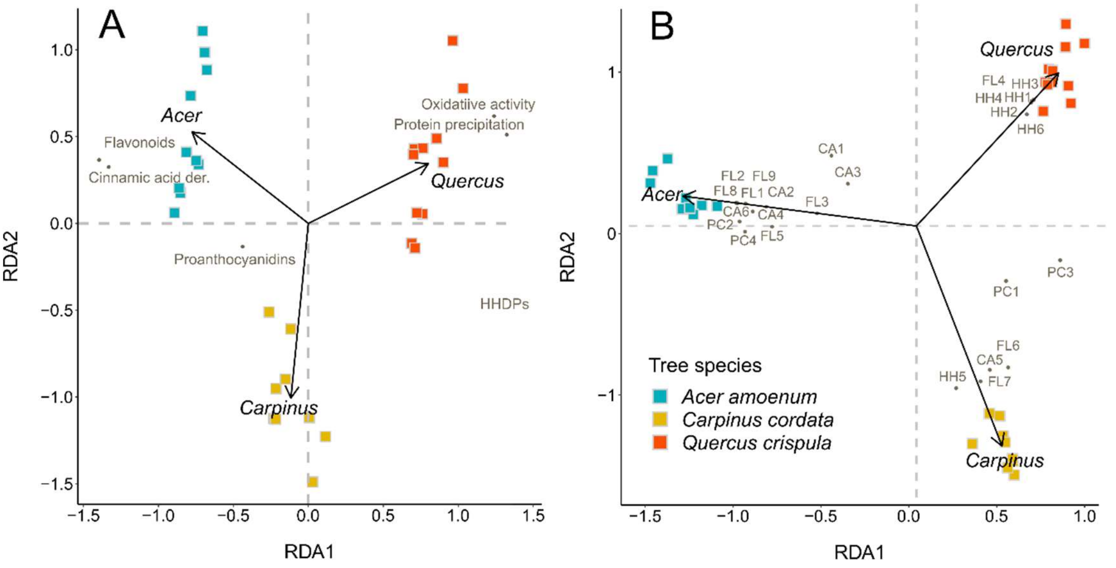

Figure S1. Variation in polyphenol subgroups and activities (A) and individual polyphenols in leaves of 30 individuals of *Acer amoenum*, *Carpinus cordata*, and *Quercus crispula* as analysed by RDA. The species identity explained 76.1% of adjusted variability in polyphenol subgroups and activities (F = 47.17, p = 0.001) and 79.2% of adjusted variability in individual polyphenols (F = 56.34, p = 0.001). Black arrows show explanatory variables, squares show leaf samples, points show individual polyphenol variables. The individual polyphenols include cinnamic acid derivatives (CA1-6), hydrolysable tannins (HH1-6), flavonoids (FL1-9), and proanthocyanidins (PC1-4). Please see Table S1 for the full list of metabolite abbreviations.

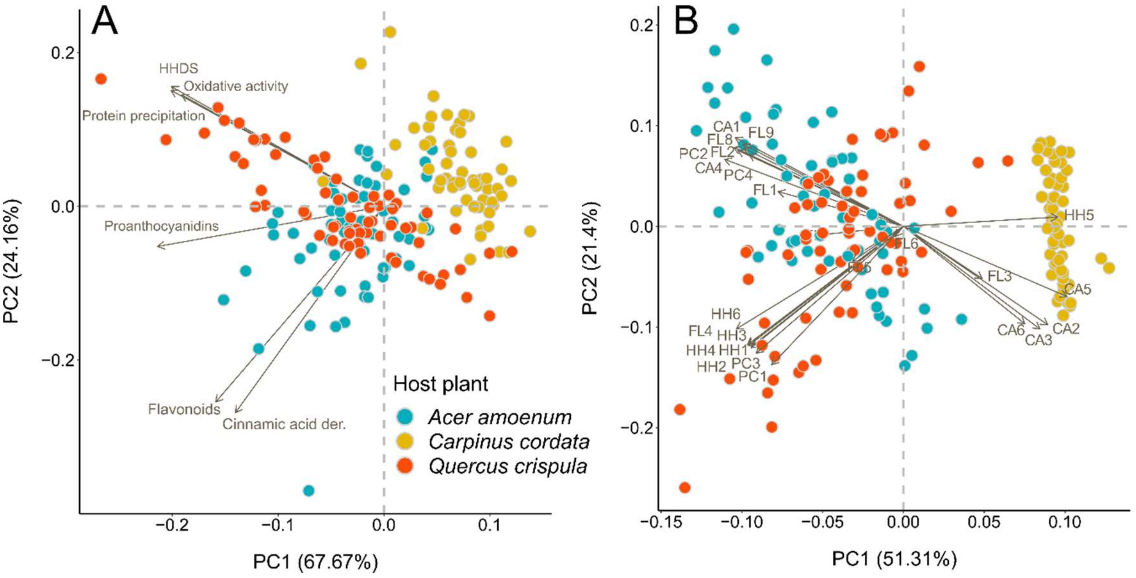

Figure S2. Variation in polyphenol subgroups and activities (A) and individual polyphenols in frass of caterpillars fed with *Acer amoenum*, *Carpinus cordata*, and *Quercus crispula* in Phase I as visualized with PCA. The first two axes explained 91.8% of variability in polyphenol subgroups and activities and 72.7% of variability in individual polyphenols. Circles show leaf samples, grey arrows show individual polyphenol variables. The individual polyphenols include cinnamic acid derivatives (CA1-6), hydrolysable tannins (HH1-6), flavonoids (FL1-9), and proanthocyanidins (PC1-4). Please see Table S1 for the full list of metabolite abbreviations.

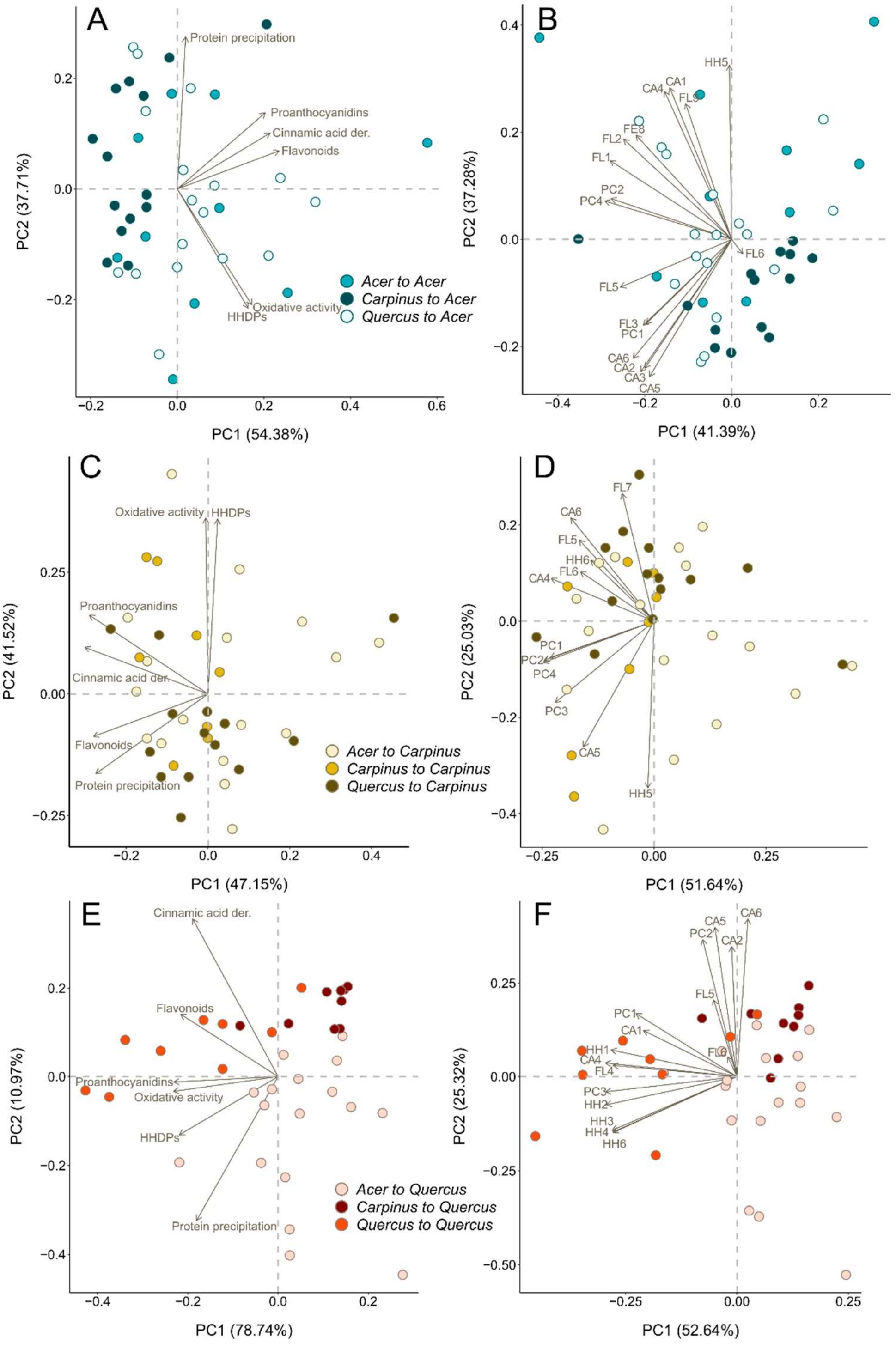

Figure S3. Variation in polyphenol subgroups and activities and individual polyphenols fed by different hosts in Phase II as visualized with PCA. In *Acer*, the first two axes explained 92.1% of variability in polyphenol subgroups and activities (A) and 78.7% of variability in individual polyphenols (B). In *Carpinus*, the first two axes explained 87.7% of variability in polyphenol subgroups and activities (C) and 76.7% of variability in individual polyphenols (D). In *Quercus*, the first two axes explained 89.7% of variability in polyphenol subgroups and activities (E) and 78.0% of variability in individual polyphenols (F). Circles show leaf samples, grey arrows show individual polyphenol variables. The individual polyphenols include cinnamic acid derivatives (CA1-6), hydrolysable tannins (HH1-6), flavonoids (FL1-9), and proanthocyanidins (PC1-4). Please see Table S1 for the full list of metabolite abbreviations.

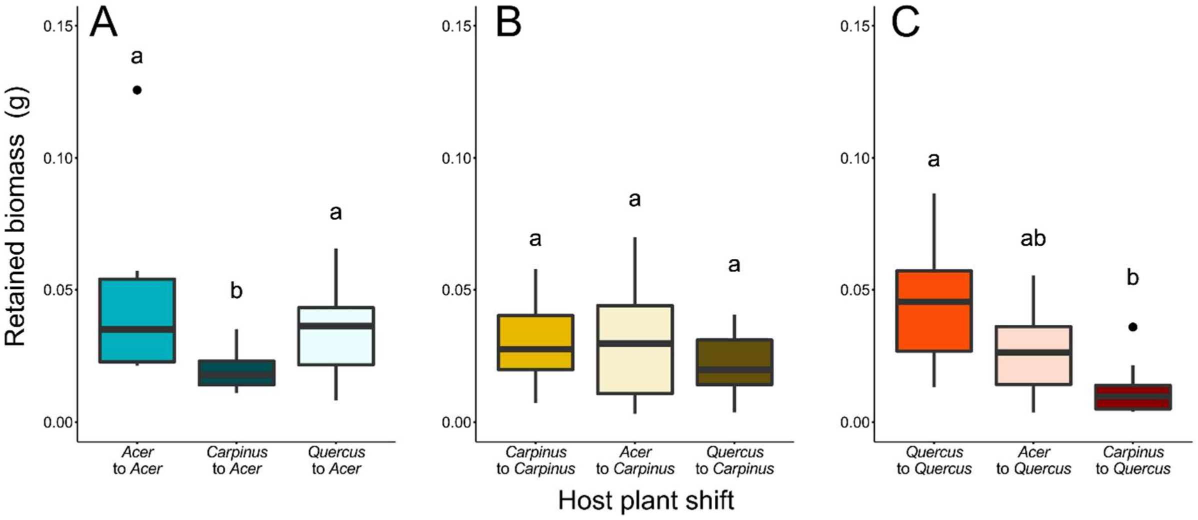

Figure S4. Biomass retained by caterpillars in Phase II. When the caterpillars were transferred to the new host in Phase II, the amount of retained biomass, significantly decreased in case of those transferred from *Carpinus* to *Acer* ($x^2_{(2)} = 11.47$ $p = 0.003$) and from *Carpinus* to *Quercus* ($x^2_{(2)} = 12.72$ $p = 0.002$) in comparison to those previously fed by those hosts. The retained biomass refers to the difference between the dry weight of leaves ingested and dry weight of frass produced.

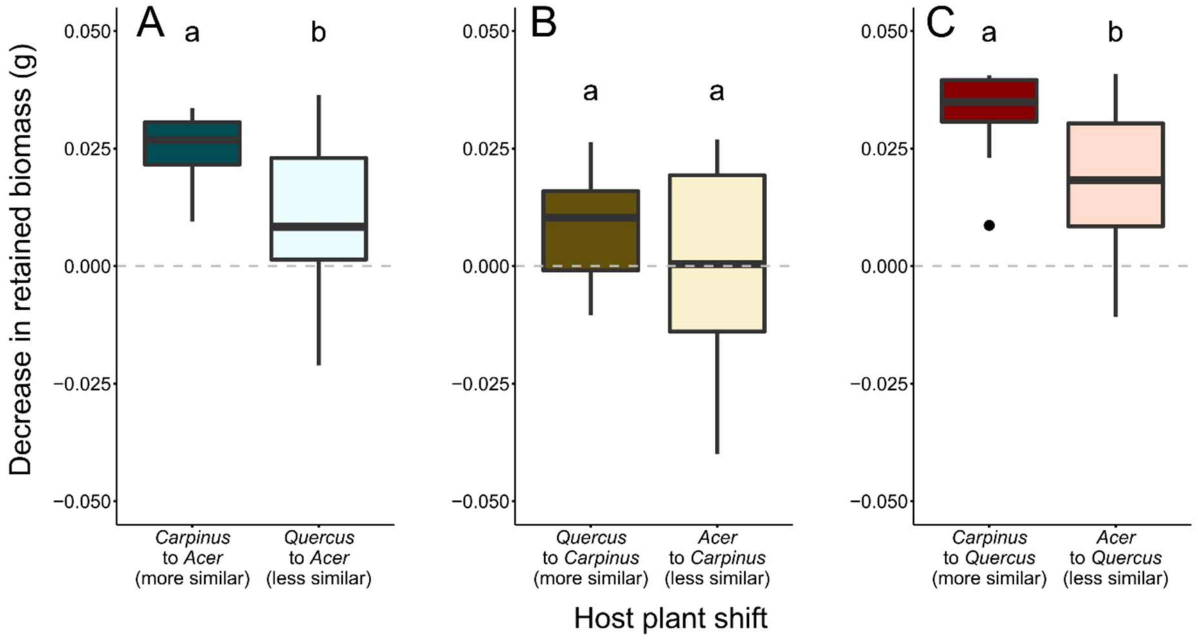

Figure S5. The relative decrease in retained biomass in caterpillars transferred to a new host. There was a smaller decrease in retained biomass in caterpillars transferred to a host less chemically similar to the original one when the similarity between hosts was measured with Bray-Curtis similarity index based on individual polyphenols in *A. amoenum* ($W = 189$, $p = 0.005$) and *Q. crispula* ($W = 120$, $p = 0.0183$). The relative decrease in retained biomass was measured as the difference between biomass retained by individual caterpillars transferred to a new host and mean biomass retained by the caterpillars fed previously by that host.
