# Supplementary material for: High intraspecific variability and previous experience affect polyphenol metabolism in polyphagous Lymantria mathura caterpillars
Source: Ecol Evol. 2024 Feb 9;14(2):e10973. doi: 10.1002/ece3.10973 (PMC10857923; doi:10.1002/ece3.10973)
Supplement: Supplementary file 2 — Table S1.–S6. [file ECE3-14-e10973-s002.docx]

**Table S1**. Concentrations of individual polyphenols, their subgroups, and activities detected in the leaves of 30 *Acer amoenum*, *Carpinus cordata*, and *Quercus* *crispula* individuals. Values show means ± SD. Traces refer to concentrations lower than 0.01 mg/g.

| **Polyphenols** | **Abbreviation** | **Retention time (min)** | **Acer leaves (mg/g)** | **Carpinus leaves (mg/g)** | **Quercus leaves (mg/g)** |
| --- | --- | --- | --- | --- | --- |
| Coumaroyl quinic acid 1 | CA1 | 2.87 | 1.91±2.6 | 0.03±0.02 | 0.72±0.69 |
| Coumaroyl quinic acid 2 | CA2 | 3.3 | 0.17±0.11 | trace | trace |
| Coumaroyl quinic acid 3 | CA3 | 3.385 | 0.02±0.02 | trace | 0.01±0.01 |
| Caffeoyl quinic acid 1 | CA4 | 2.50 | 39.8±11.99 | 2.92±1.46 | 3.36±3.09 |
| Caffeoyl quinic acid 2 | CA5 | 2.95 | 0.09±0.07 | 8.81±4.16 | 0.38±0.42 |
| Caffeoyl quinic acid 3 | CA6 | 3.04 | 11.48±7.54 | trace | 0.01±0.02 |
| Vescavaloninic acid | HH1 | 2.00 | trace | trace | 8.74±2.52 |
| Vescalagin | HH2 | 2.25 | trace | trace | 11.41±4.07 |
| Castalagin | HH3 | 2.60 | 0.01±0.03 | trace | 6.38±2.45 |
| Cocciferin | HH4 | 3.30 | trace | trace | 8.73±2.28 |
| Geraniin | HH5 | 3.41 | 0.4±0.13 | 11.68±2.84 | 0.01±0.02 |
| Monogalloylglucose | HH6 | 1.26 | 0.03±0.03 | 0.06±0.11 | 0.91±0.38 |
| Kaempferol glycoside 1 | FL1 | 3.58 | 6.78±1.6 | trace | trace |
| Kaempferol glycoside 2 | FL2 | 3.75 | 4.39±1.04 | trace | trace |
| Kaempferol glycoside 3 | FL3 | 4.31 | 0.05±0.07 | trace | trace |
| Kaempferol galloylglycoside | FL4 | 4.30 | trace | trace | 2.22±0.81 |
| Quercetin glycoside | FL5 | 4.05 | 5.7±2.21 | 2.17±1.44 | 1.59±0.47 |
| Quercetin arabinoside | FL6 | 4.34 | 0.14±0.08 | 2.78±0.45 | 0.44±0.16 |
| Quercetin rhamnoside | FL7 | 4.45 | trace | 6.24±1.39 | 0.04±0.09 |
| Apigenin galactoside | FL8 | 4.01 | 16.39±3.15 | 0.01±0.01 | 0.01±0.01 |
| Apigenin glucoside | FL9 | 4.01 | 13.35±4.01 | trace | trace |
| Catechin | PC1 | 2.89 | 0.38±0.72 | 2.08±1.9 | 0.98±0.52 |
| Epicatechin | PC2 | 3.335 | 2.11±0.43 | 0.17±0.25 | trace |
| PC dimer 1 | PC3 | 2.87 | 0.01±0.01 | 4.44±3.28 | 3.58±2.94 |
| PC dimer 2 | PC4 | 3.25 | 4.46±0.85 | 0.57±0.97 | 0.02±0.02 |
| Total cinnamic acid derivatives | - | - | 53.46±13.56 | 11.75±5.3 | 4.48±2.77 |
| Total flavonoids | - | - | 49.32±5.49 | 13.52±4.43 | 6.2±1.3 |
| Total hydrolysable tannins | - | - | 0.45±0.14 | 11.75±2.86 | 36.18±7.73 |
| Total proanthocyanidins | - | - | 4.46±0.85 | 5.01±3.38 | 3.59±2.94 |
| Oxidative activity | - | - | 14.64±3.19 | 13.49±2.36 | 41.3±10.11 |
| **Row Labels** | **Abbreviation** | **Retention time (min)** | **Acer leaves (mg/g)** | **Carpinus leaves (mg/g)** | **Quercus leaves (mg/g)** |
| Protein precipitation capacity | - | - | 16.24±4.19 | 17.06±3.22 | 60.56±7.57 |

**Table S2**. Concentrations of individual polyphenols, their subgroups, and activities detected in the leaves used for feeding of caterpillars and in the resulting frass in Phase I. Values for leaves show means ± SD calculated based on leaves collected immediately at the beginning of Phase I, and after 8, 16, and 24 hours. Values for frass show means± SD based on values from frass produced by individual caterpillars. Traces refer to concentrations lower than 0.01 mg/g.

| **Polyphenols** | **Abbreviation** | **Retention time (min)** | **Acer leaves (mg/g)** | **Acer frass**  **(mg/g)** | **Carpinus leaves (mg/g)** | **Carpinus frass (mg/g)** | **Quercus leaves (mg/g)** | **Quercus frass (mg/g)** |
| --- | --- | --- | --- | --- | --- | --- | --- | --- |
| Coumaroyl quinic acid 1 | CA1 | 2.87 | 6.8±0.42 | 3.73±0.58 | 0.01±0.01 | 0.01±0.03 | 0.22±0.02 | 0.1±0.03 |
| Coumaroyl quinic acid 2 | CA2 | 3.3 | 0.11±0.02 | 0.86±0.15 | trace | trace | trace | 0.02±0.01 |
| Coumaroyl quinic acid 3 | CA3 | 3.385 | 0.01±0.00 | 0.69±0.15 | trace | trace | trace | trace |
| Caffeoyl quinic acid 1 | CA4 | 2.50 | 18.82±0.59 | 10.23±1.63 | 1.06±0.45 | 1.28±0.87 | 3.11±0.18 | 1.13±0.35 |
| Caffeoyl quinic acid 2 | CA5 | 2.95 | 0.01±0.01 | 5.52±1.17 | 2.91±1.66 | 1.3±0.89 | 0.47±0.17 | 0.71±0.24 |
| Caffeoyl quinic acid 3 | CA6 | 3.04 | 1.30±0.14 | 7.56±1.4 | trace | 0.81±0.62 | trace | 0.65±0.21 |
| Vescavaloninic acid | HH1 | 2.00 | trace | trace | 0.01±0.02 | trace | 7.08±0.98 | 2.17±0.98 |
| Vescalagin | HH2 | 2.25 | 0.02±0.03 | trace | 0.01±0.01 | trace | 11.71±1.71 | 3.04±1.2 |
| Castalagin | HH3 | 2.60 | 0.01±0.01 | trace | 0.02±0.02 | trace | 10.42±0.32 | 1.91±0.6 |
| Cocciferin | HH4 | 3.30 | trace | trace | trace | trace | 3.72±0.12 | 0.48±0.22 |
| Geraniin | HH5 | 3.41 | 1.34±0.30 | 0.05±0.03 | 7.89±2.70 | 0.15±0.07 | 0.01±0.02 | trace |
| Monogalloylglucose | HH6 | 1.26 | 0.03±0.01 | trace | trace | 0.01±0.01 | 0.43±0.13 | 0.07±0.03 |
| Kaempferol glycoside 1 | FL1 | 3.58 | 3.71±0.51 | 4.47±0.53 | trace | 0.01±0.04 | trace | trace |
| Kaempferol glycoside 2 | FL2 | 3.75 | 2.90±0.42 | 2.87±0.4 | trace | trace | trace | trace |
| Kaempferol glycoside 3 | FL3 | 4.31 | trace | 0.18±0.2 | trace | trace | trace | trace |
| Kaempferol galloylglycoside | FL4 | 4.30 | trace | trace | trace | trace | 1.63±0.16 | 0.61±0.32 |
| Quercetin glycoside | FL5 | 4.05 | 2.79±0.66 | 4.44±1.99 | 0.68±0.17 | 1.39±0.76 | 0.98±0.14 | 0.99±0.37 |
| Quercetin arabinoside | FL6 | 4.34 | 0.07±0.01 | 0.13±0.06 | 1.86±0.21 | 2.4±0.63 | 0.29±0.01 | 0.32±0.07 |
| Quercetin rhamnoside | FL7 | 4.45 | trace | trace | 2.33±0.49 | 4.23±1.31 | trace | trace |
| Apigenin galactoside | FL8 | 4.01 | 12.96±0.51 | 12.64±1.14 | trace | 0.04±0.29 | 0.01±0.01 | trace |
| Apigenin glucoside | FL9 | 4.01 | 11.66±0.44 | 10.92±2.09 | trace | 0.01±0.09 | trace | trace |
| Catechin | PC1 | 2.89 | 0.03±0.02 | 0.11±0.06 | 0.17±0.12 | 0.02±0.05 | 1.62±0.95 | 0.36±0.22 |
| Epicatechin | PC2 | 3.335 | 2.46±0.21 | 1.53±0.61 | 0.04±0.04 | 0.01±0.02 | trace | 0.01±0.01 |
| PC dimer 1 | PC3 | 2.87 | trace | trace | 0.36±0.22 | 0.03±0.1 | 4.79±0.97 | 0.58±0.35 |
| PC dimer 2 | PC4 | 3.25 | 6.00±0.36 | 5.22±1.31 | 0.23±0.19 | 0.04±0.07 | trace | trace |
| Total cinnamic acid derivatives | - | - | 27.04±0.14 | 28.6±4.5 | 3.98±2.04 | 3.4±2.36 | 3.79±0.27 | 2.61±0.79 |
| Total flavonoids | - | - | 36.61±1.01 | 37.3±2.86 | 5.10±0.91 | 8.14±2.54 | 4.96±1.09 | 2.35±0.79 |
| Total hydrolysable tannins | - | - | 1.41±0.34 | 0.06±0.03 | 7.93±2.74 | 0.17±0.08 | 33.37±2.49 | 7.68±2.71 |
| Total proanthocyanidins | - | - | 6.01±0.36 | 5.22±1.31 | 0.59±0.39 | 0.07±0.16 | 4.79±0.97 | 0.58±0.35 |
| Oxidative activity | - | - | 12.6±1.77 | 8.92±5.27 | 12.7±0.92 | 3.56±2.2 | 34.45±4.15 | 16.29±5.94 |
| **Row Labels** | **Abbreviation** | **Retention time (min)** | **Acer leaves (mg/g)** | **Acer frass**  **(mg/g)** | **Carpinus leaves (mg/g)** | **Carpinus frass (mg/g)** | **Quercus leaves (mg/g)** | **Quercus frass (mg/g)** |
| Protein precipitation capacity | - | - | 20.68±2.10 | 27.87±4.73 | 9.38±3.14 | 0.21±0.87 | 52.44±2.66 | 26.43±6.26 |

**Table S3**. Concentrations of individual polyphenols, their subgroups, and activities detected in the *Carpinus* leaves used for feeding of caterpillars and in the resulting frass in Phase II. Values for leaves show means ± SD calculated based on leaves collected immediately at the beginning of Phase II, and after 8, 16, and 24 hours. Values for frass show means± SD based on values from frass produced by individual caterpillars. Traces refer to concentrations lower than 0.01 mg/g. In Phase II, we considered only the metabolites that we previously recorded (>0.01 mg/g) in more than 25% frass samples from *Carpinus* in Phase I.

| **Polyphenols** | **Abbreviation** | **Retention time (min)** | **Carpinus leaves**  **(mg/g)** | **Carpinus to Carpinus frass**  **(mg/g)** | **Acer to Carpinus frass**  **(mg/g)** | **Quercus to Carpinus frass**  **(mg/g)** |
| --- | --- | --- | --- | --- | --- | --- |
| Caffeoyl quinic acid 1 | CA4 | 2.87 | 2.58±0.87 | 2.29±1.18 | 4.54±1.64 | 3.21±1.89 |
| Caffeoyl quinic acid 2 | CA5 | 3.3 | 8.86±2.41 | 2.5±1.18 | 4.65±1.67 | 3.18±1.56 |
| Caffeoyl quinic acid 3 | CA6 | 3.385 | trace | 1.61±0.89 | 3.37±1.30 | 2.21±1.48 |
| Geraniin | HH5 | 3.41 | 7.17±0.45 | 0.24±0.10 | 0.23±0.10 | 0.16±0.06 |
| Monogalloylglucose | HH6 | 1.26 | 0.01±0.01 | 0.02±0.01 | 0.02±0.01 | 0.02±0.01 |
| Quercetin glycoside | FL5 | 4.05 | 1.50±0.44 | 2.09±0.79 | 2.61±0.87 | 1.87±0.75 |
| Quercetin arabinoside | FL6 | 4.34 | 2.62±0.41 | 2.97±0.63 | 3.04±0.29 | 2.75±0.54 |
| Quercetin rhamnoside | FL7 | 4.45 | 2.88±0.73 | 5.21±1.79 | 4.73±1.10 | 4.30±1.28 |
| Catechin | PC1 | 2.89 | 1.24±0.40 | 0.36±0.33 | 0.61±0.65 | 0.55±0.86 |
| Epicatechin | PC2 | 3.335 | 0.39±0.16 | 0.11±0.08 | 0.22±0.19 | 0.15±0.22 |
| PC dimer 1 | PC3 | 2.87 | 2.68±0.73 | 0.49±0.42 | 0.93±1.03 | 0.69±1.00 |
| PC dimer 2 | PC4 | 3.25 | 1.65±0.39 | 0.49±0.33 | 0.98±0.73 | 0.61±0.69 |
| Total cinnamic acid derivatives | S1 | - | 11.45±3.08 | 6.41±3.21 | 12.56±4.55 | 8.60±4.90 |
| Total flavonoids | S2 | - | 8.62±1.50 | 10.76±2.96 | 11.23±2.27 | 9.63±2.69 |
| Total hydrolysable tannins | S3 | - | 7.17±0.45 | 0.25±0.10 | 0.25±0.10 | 0.17±0.06 |
| Total proanthocyanidins | S4 | - | 4.33±1.10 | 0.99±0.72 | 1.91±1.72 | 1.30±1.67 |
| Oxidative activity (mg/g) | S5 | - | 16.45±2.41 | 5.26±2.70 | 8.18±2.62 | 5.89±2.62 |
| Protein precipitation capacity (mg/g) | S6 | - | 15.11±3.18 | 13.04±6.79 | 11.49±6.06 | 14.35±9.63 |

**Table S4**. Concentrations of individual polyphenols, their subgroups, and activities detected in the *Quercus* leaves used for feeding of caterpillars and in the resulting frass in Phase II. Values for leaves show means ± SD calculated based on leaves collected immediately at the beginning of Phase II, and after 8, 16, and 24 hours. Values for frass show means± SD based on values from frass produced by individual caterpillars. Traces refer to concentrations lower than 0.01 mg/g. In Phase II, we considered only the metabolites that we previously recorded (>0.01 mg/g) in more than 25% frass samples from *Quercus* in Phase I.

| **Polyphenols** | **Abbreviation** | **Retention time**  **(min)** | **Quercus leaves**  **(mg/g)** | **Quercus to Quercus frass**  **(mg/g)** | **Acer to Quercus frass**  **(mg/g)** | **Carpinus to Quercus frass**  **(mg/g)** |
| --- | --- | --- | --- | --- | --- | --- |
| Coumaroyl quinic acid 1 | CA1 | 2.87 | 0.22±0.02 | 0.12±0.01 | 0.45±0.29 | 0.08±0.03 |
| Coumaroyl quinic acid 2 | CA2 | 3.30 | trace | 0.02±0.01 | 0.11±0.07 | 0.02±0.01 |
| Caffeoyl quinic acid 1 | CA4 | 2.50 | 3.06±0.71 | 1.28±0.40 | 2.70±0.91 | 1.66±0.73 |
| Caffeoyl quinic acid 2 | CA5 | 2.95 | 0.31±0.08 | 0.78±0.34 | 1.80±0.64 | 0.94±0.45 |
| Caffeoyl quinic acid 3 | CA6 | 3.04 | trace | 0.70±0.21 | 1.59±0.53 | 0.88±0.40 |
| Vescavaloninic acid | HH1 | 2.00 | 5.81±0.70 | 2.39±1.28 | 3.92±2.19 | 1.76±0.93 |
| Vescalagin | HH2 | 2.25 | 10.79±1.26 | 3.30±1.10 | 4.85±2.05 | 2.35±1.32 |
| Castalagin | HH3 | 2.60 | 9.24±0.25 | 1.90±0.45 | 2.66±0.73 | 1.98±0.84 |
| Cocciferin | HH4 | 3.30 | 3.32±0.52 | 0.44±0.18 | 0.82±0.36 | 0.54±0.24 |
| Monogalloylglucose | HH6 | 1.26 | 0.35±0.04 | 0.06±0.02 | 0.08±0.04 | 0.06±0.03 |
| Kaempferol galloylglycoside | FL4 | 4.30 | 1.21±0.23 | 0.55±0.31 | 0.85±0.49 | 0.58±0.47 |
| Quercetin glycoside | FL5 | 4.05 | 0.79±0.09 | 1.09±0.27 | 1.27±0.44 | 0.89±0.31 |
| Quercetin arabinoside | FL6 | 4.34 | 0.24±0.03 | 0.35±0.05 | 0.31±0.07 | 0.54±0.14 |
| Catechin | PC1 | 2.89 | 1.20±0.49 | 0.39±0.26 | 0.86±0.91 | 0.35±0.41 |
| Epicatechin | PC2 | 3.335 | trace | 0.01±0.01 | 0.04±0.03 | 0.01±0.01 |
| PC dimer 1 | PC3 | 2.87 | 4.40±1.00 | 0.60±0.41 | 1.27±1.15 | 0.53±0.58 |
| Total cinnamic acid derivatives | S1 | - | 3.59±0.78 | 2.90±0.94 | 6.65±2.25 | 3.59±1.58 |
| Total flavonoids | S2 | - | 3.78±0.61 | 2.43±0.62 | 3.40±1.39 | 2.42±1.02 |
| Total hydrolysable tannins | S3 | - | 29.52±1.70 | 5.70±1.56 | 8.41±3.01 | 4.92±2.31 |
| Total proanthocyanidins | S4 | - | 4.40±1.00 | 0.60±0.41 | 1.27±1.15 | 0.53±0.58 |
| Oxidative activity (mg/g) | S5 | - | 32.60±0.75 | 14.87±5.16 | 22.32±7.70 | 12.78±5.69 |
| Protein precipitation capacity (mg/g) | S6 | - | 56.30±2.22 | 54.61±8.90 | 38.25±7.81 | 50.8±21.26 |

**Table S5**. Concentrations of individual polyphenols, their subgroups, and activities detected in the *Acer* leaves used for feeding of caterpillars and in the resulting frass in Phase II. Values for leaves show means ± SD calculated based on leaves collected immediately at the beginning of Phase II, and after 8, 16, and 24 hours. Values for frass show means± SD based on values from frass produced by individual caterpillars. Traces refer to concentrations lower than 0.01 mg/g. In Phase II, we considered only the metabolites that we previously recorded (>0.01 mg/g) in more than 25% frass samples from *Acer* in Phase I.

| **Polyphenols** | **Abbreviation** | **Retention time**  **(min)** | **Acer leaves**  **(mg/g)** | **Acer to Acer frass**  **(mg/g)** | **Carpinus to Acer frass**  **(mg/g)** | **Quercus to Acer frass**  **(mg/g)** |
| --- | --- | --- | --- | --- | --- | --- |
| Coumaroyl quinic acid 1 | CA1 | 2.87 | 7.12±0.23 | 4.20±0.91 | 4.02±1.20 | 4.04±0.73 |
| Coumaroyl quinic acid 2 | CA2 | 3.30 | 0.11±0.01 | 0.90±0.21 | 0.87±0.26 | 0.88±0.16 |
| Coumaroyl quinic acid 3 | CA3 | 3.385 | 0.01±0.00 | 0.88±0.30 | 0.84±0.28 | 0.81±0.19 |
| Caffeoyl quinic acid 1 | CA4 | 2.25 | 19.72±1.46 | 11.76±2.86 | 11.54±3.02 | 11.19±2.36 |
| Caffeoyl quinic acid 2 | CA5 | 2.95 | 0.01±0.01 | 5.99±1.90 | 6.01±1.84 | 5.52±1.46 |
| Caffeoyl quinic acid 3 | CA6 | 3.04 | 1.37±0.14 | 8.52±2.33 | 8.99±2.56 | 8.11±2.04 |
| Geraniin | HH5 | 3.41 | 1.39±0.52 | 0.04±0.02 | 0.04±0.04 | 0.05±0.03 |
| Kaempferol glycoside 1 | FL1 | 3.58 | 4.12±0.10 | 4.77±0.83 | 4.96±1.45 | 4.58±0.69 |
| Kaempferol glycoside 2 | FL2 | 3.75 | 2.99±0.15 | 3.32±0.53 | 3.34±1.00 | 3.18±0.47 |
| Kaempferol glycoside 3 | FL3 | 4.31 | trace | 0.29±0.41 | 0.22±0.35 | 0.12±0.24 |
| Quercetin glycoside | FL5 | 4.05 | 2.83±0.46 | 4.94±2.47 | 5.26±2.85 | 3.72±2.02 |
| Quercetin arabinoside | FL6 | 4.34 | 0.06±0.02 | 0.16±0.09 | 0.51±0.67 | 0.14±0.06 |
| Apigenin galactoside | FL8 | 4.01 | 12.26±0.82 | 13.4±1.62 | 13.31±3.81 | 14.27±1.23 |
| Apigenin glucoside | FL9 | 4.01 | 12.04±1.55 | 12.13±2.49 | 12.66±4.45 | 13.48±2.45 |
| Catechin | PC1 | 2.89 | 0.02±0.01 | 0.18±0.08 | 0.22±0.12 | 0.17±0.10 |
| Epicatechin | PC2 | 3.335 | 2.11±0.22 | 2.09±0.93 | 2.04±0.93 | 1.58±0.73 |
| PC dimer 2 | PC4 | 3.25 | 5.49±0.37 | 6.40±1.76 | 6.27±2.22 | 5.41±1.54 |
| Total cinnamic acid derivatives | S1 | - | 28.33±1.79 | 32.25±8.28 | 32.26±8.75 | 30.55±6.04 |
| Total flavonoids | S2 | - | 36.48±1.53 | 41.26±4.76 | 42.52±10.95 | 41.24±4.66 |
| Total hydrolysable tannins | S3 | - | 1.47±0.50 | 0.04±0.02 | 0.04±0.04 | 0.05±0.03 |
| Total proanthocyanidins | S4 | - | 5.50±0.38 | 6.40±1.76 | 6.27±2.22 | 5.41±1.54 |
| Oxidative activity (mg/g) | S5 | - | 14.05±2.00 | 10.42±4.34 | 9.21±4.40 | 6.11±4.91 |
| Protein precipitation capacity (mg/g) | S6 | - | 25.60±2.84 | 30.59±6.28 | 50.68±12.68 | 49.59±13.27 |

**Table S6.** Bray–Curtis dissimilarity based on the presence and concentration of individual polyphenols or total contents of polyphenol subgroups and activities in leaves used for feeding in Phase II.

|  | **Individual**  **polyphenols** | **Polyphenol subgroups**  **and activities** |
| --- | --- | --- |
| *Acer* and *Carpinus* | 0.9050388 | 0.5406585 |
| *Acer* and *Quercus* | 0.9446669 | 0.4399541 |
| *Carpinus* and *Quercus* | 0.8458122 | 0.6033478 |
